# Acupuncture on treating angina pectoris: A systematic review

**DOI:** 10.1097/MD.0000000000018548

**Published:** 2020-01-10

**Authors:** Ji-sheng Wang, Xu-dong Yu, Sheng Deng, Hong-wei Yuan, Hai-song Li

**Affiliations:** a Department of Andrology; b Department of Acupuncture and Moxibustion, Dongzhimen Hospital, Beijing University of Chinese Medicine, Beijing, China.

**Keywords:** acupuncture, angina pectoris, systematic review

## Abstract

**Background:**

Coronary heart disease angina pectoris is a common clinical symptom in patients with coronary heart disease, due to coronary atherosclerotic stenosis or sputum leading to coronary insufficiency, myocardial transient ischemia, hypoxia caused by precordial pain as the main clinical manifestations Group syndrome. Coronary heart disease angina causes coronary blood flow insufficiency, cannot meet the normal activities of myocardial cells, leading to myocardial ischemia or necrosis. When the disease occurs, there is paroxysmal and crushing pain in the precordial area of the patient. Therefore, we recognize the importance of the disease and have paid enough attention. Clinical studies in recent years have found that the use of acupuncture in the treatment of angina pectoris has a good clinical application prospect. This study was conducted to study the effect of using acupuncture to treat angina pectoris.

**Methods and analysis:**

We will search for PubMed, Cochrane Library, AMED, EMbase, WorldSciNet, Nature, Science online and China Journal Full-text Database, China Biomedical Literature CD-ROM Database (CBM), and related randomized controlled trials included in the China Resources Database. The time is limited from the construction of the library to November 2019. We will use the criteria provided by Cochrane 5.1.0 for quality assessment and risk assessment of the included studies, and use the Revman 5.3 and Stata13.0 software for meta-analysis of the effectiveness, recurrence rate, and symptom scores of angina pectoris.

**Ethics and dissemination:**

This systematic review will evaluate the efficacy and safety of acupuncture for angina pectoris. Because all of the data used in this systematic review and meta-analysis have been published, this review does not require ethical approval. Furthermore, all data will be analyzed anonymously during the review process Trial.

**Registration number:**

PROSPERO CRD42019138003.

## 1 Introduction

### 1.1 Background

Angina pectoris is a clinical syndrome characterized by insufficient blood supply to the coronary arteries, sudden myocardial ischemia, and hypoxia, with episodic chest pain or chest discomfort.^[1,2]^ Angina is the pain felt by the heart's ischemic reflex to the surface of the body. It is characterized by paroxysmal anterior chest and compression pain.^[3,4]^ It can be accompanied by other symptoms. The pain is mainly located in the back of the sternum and can be radiated to the anterior and left upper limbs.^[5]^ Or emotional excitement often occurs, each episode lasts for 3 to 5 minutes, can be done once a few days, or several times a day, rest or disappear with nitrate preparations.^[6–8]^ The disease is more common in men, most of them older than 40 years, tired, emotional, full of food, cold, rainy weather, acute circulatory failure, among others,^[10]^ are common causes. There are many classification methods for angina pectoris, which are not unified at home and abroad.^[11]^ In recent years, angina pectoris has been classified into 3 categories according to the World Health Organization's “Name and Diagnostic Criteria for Ischemic Heart Disease.”^[12]^ One type is exertional angina pectoris, which is divided into first-onset angina pectoris, stable angina pectoris, and worsening angina pectoris, also known as progressive angina or unstable angina; the second type is spontaneous angina pectoris, which is divided into lying Type angina pectoris, variant angina pectoris, intermediate syndrome, post-infarction angina pectoris; 3 types of mixed angina pectoris.^[13]^

The direct cause of angina pectoris is the absolute or relative deficiency of myocardial blood supply; therefore, various reductions in myocardial blood (blood oxygen) supply (such as intravascular thrombosis, vasospasm) and increased oxygen consumption (such as exercise, increased heart rate) factors can induce angina.^[14]^ Myocardial blood supply is mainly due to coronary heart disease. Sometimes, other types of heart disease or uncontrolled high blood pressure can also cause angina. There is a need to confirm the diagnosis by x-ray, electrocardiogram, selective coronary angiography.^[15]^

Acupuncture is an integral part of traditional Chinese medicine. According to reports in the literature, acupuncture can relieve angina symptoms and reduce the frequency of angina and pain intensity in patients with angina pectoris.^[16]^ After preliminary searches and database analysis, we found that the frequency of randomized controlled trials of acupuncture for angina pectoris was increasing.^[17,18]^ Previous clinical trials have shown that acupuncture can relieve symptoms and improve the quality of life of patients.^[19]^ These effects persist in angina pectoris patients. However, due to the size of the clinical center and the limited number of samples, the current level of evidence-based medical evidence is still insufficient. Therefore, we hope to evaluate the effectiveness and safety of acupuncture in the treatment of angina pectoris through meta-analysis, and provide a sufficient basis for its clinical application.^[20]^

## 2 Methods

This systematic review protocol has been registered on PROSPERO CRD42019138003 (https://www.crd.york.ac.uk/prospero/display_record.php?RecordID=138003). The protocol follows the Cochrane Handbook for Systematic Reviews of Interventions and the Preferred Reporting Items for Systematic Reviews and Meta-Analysis Protocol statement guidelines. We will describe the changes in our full review if needed.

### 2.1 Inclusion criteria for study selection

#### 2.1.1 Types of studies

We will gather all studies of acupuncture on treating angina pectoris: a systematic review and meta-analysis which, no matter whether they have been published or not, base on the method of RCT (randomized controlled trial). The language is limited to Chinese and English. Non-RCTs quasi-RCTs, series of case reports, and cross research will be excluded.

#### 2.1.2 Types of participants

Our inclusion criteria are: meet the clinical diagnostic criteria for angina; comply with ECG and Holter diagnosis. Both the patient and the family informed the study and signed a consent form.

#### 2.1.3 Types of interventions

We will adopt acupuncture treatment of angina pectoris as experimental interventions. Considering that the theory of pharmaco-acupuncture and point injection belongs to another part of TCM, so they will be considered for exclusion.

##### 2.1.3.1 Control interventions

As for control intervention, a person receiving virtual acupuncture treatment can be used as a placebo control, or as a blank control without receiving any treatment. However, once they receive acupuncture-combined drugs or other Chinese medicine, the trial will be rejected.

The following treatment comparisons will be studied:

1. Acupuncture and no treatment
2. Acupuncture and placebo/false acupuncture
3. Acupuncture and drug treatment
4. Acupuncture and other active therapies
5. Acupuncture combined with another active therapy compared to the same treatment alone.

#### 2.1.4 Types of outcome measures

The main criteria are: symptoms completely disappear; the number and duration of angina attacks decreased by more than 80%; the ECG is back to normal.

##### 2.1.4.1 Secondary outcomes

Secondary assessment criteria include: the number of angina pectoris is significantly reduced. At the same time, close attention should be paid to whether adverse reactions or adverse events occur during the experiment to comprehensively evaluate the clinical efficacy and safety of acupuncture in the treatment of angina pectoris.

#### 2.1.5 Electronic searches

Database search includes: Search PubMed, Cochrane, Library, AMED, EMbase, WorldSciNet; Nature Science online and China National Knowledge Infrastructure, China Biology Medicine disc (CBMdisc). The temporal interval is limited from the time that the databases created to November 2019, and the combination of keyword and free word retrieval is adopted. The search terms include “acupuncture,” “skin needle,” “dermal needle,” “angina pectoris,” and “coronary heart disease angina pectoris.” The search term in the Chinese database is the translation of the above word. The complete PubMed search strategy is summarized in Table 1.

**Table 1:**
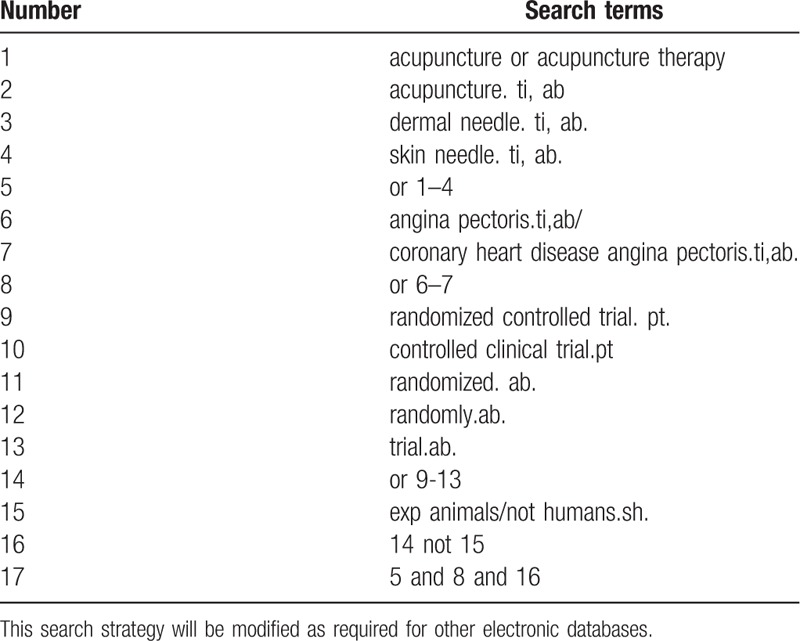
Search strategy used in PubMed database.

#### 2.1.6 Searching other resources

The manual search mainly searched for relevant literatures, earlier than the database above-mentioned, such as “China Rehabilitation Medicine Journal,” “Chinese Acupuncture,” “Chinese Journal of Physical Medicine and Rehabilitation,” “Acupuncture Clinical Journal,” and “Shanghai Acupuncture Journal.”

### 2.2 Data collection and analysis

#### 2.2.1 Study identification

**Figure 1:**
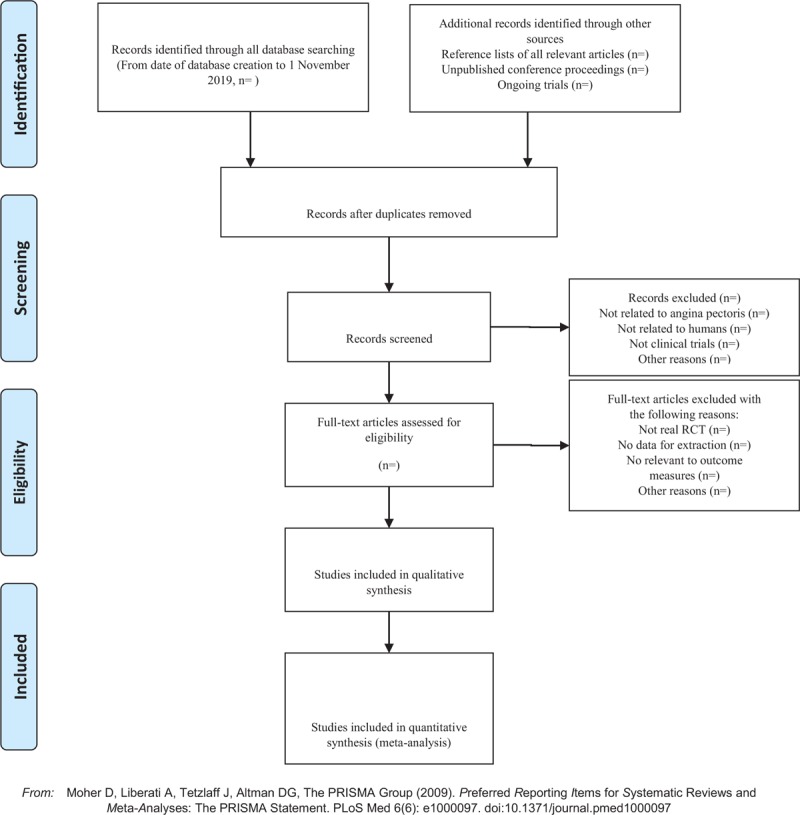
The PRISMA flow chart.

1. There are 2 researchers filtering out the literature that clearly do not conform to the study such as meeting minutes dissertations reviews animal experiments and so on, which, after excluding all the retrieved documents from the duplicated literature, adopt the method of reading the title of the literature abstracts, among others. The details of selection process will be shown in the PRISMA flow chart (Fig. 1).
2. The second time of screening the literature: skimming the remaining documents and filtering out unqualified documents such as case reports theoretical discussions and nonconformance of interventions.
3. The third time of screening the literature: carefully reading the remaining documents and strictly filtering out unqualified documents such as general controlled trials, lacking control group, deficiency of random allocation, incompatible outcome indicator and the appearance of similar data, among others
4. As for the literature that cannot be ensured, it would be confirmed by the discussion of the 2 researchers. And if they cannot reach an agreement, the third-party experts would get involved, which aims at absorbing the appropriate RCTs into the study.

#### 2.2.2 Data extraction and management

The literature data extraction will be completed independently by 2 researchers and the data form uniformly developed by the researcher was filled out. The data extraction content includes the following:

(1) General information: article title, first author, corresponding author, time of publication research, evaluation correspondence, contact information.
(2) Research method: design pattern, ample size, random allocation, random hiding, blind method, baseline level.
(3) Participants: patients’ age, sex, angina pectoris diagnostic criteria, severity, ethnicity study, location.
(4) Intervention: acupuncture, acupuncture point, period of treatment, treatment frequency.
(5) Efficacy evaluation: main observation indicators secondary observation indicators safety indicators and number of adverse reactions.
(6) Note: sources of funds, medical ethics audit, important references.

#### 2.2.3 Assessment of risk of bias in included studies

As for the Literature quality evaluation, we will use the bias risk assessment tool recommended by Cochrane to assess the quality of all included literature and risk of bias (ROB). The assessment includes: sequence generation; allocation concealment; blinding of participants, personnel, and outcome assessors; incomplete outcome data; selective outcome reporting; other sources of bias. The evaluation above would be independently evaluated by 2 researchers. If there are different opinions, we discuss them. If there are still differences exist, we would consult the third appraiser. Otherwise, we need to consult with the Cochrane Professional Group for solution.

### 2.3 Statistical analysis

The meta-analysis studied in this review will adopt Rev Man5.3 and Stata13.0 statistical software. Heterogeneity test will be used for the inclusion of the study, and random- or fixed-effect models will be adopted, with *P* < .05 as the test standard. If the heterogeneity between the results is too large, the random-effects model, which deduces the source of heterogeneity by sensitivity analysis, will be used for the rest analysis. Secondly, according to the different type of statistical data, the binary categorical variable will use the odds ratio (OR) and its 95% confidence interval (CI) as the effect analysis index. As for the continuous variable, the standardized mean difference and its 95% CI will be used as the effect analysis index. If the outcome measures only provide the means and standards deviation before or after treatment, the Mean_change_ and the SD_change_ are obtained according to the method provided in Cochrane Handbook 5.1.0: 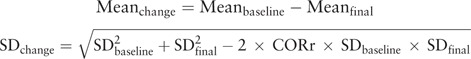


The forest map and funnel plot were drawn and analyzed using Rev Man5.3 software, and the funnel plot was used to analyze potential publication bias. As for the literature quality evaluation, we will use the bias risk assessment tool recommended by Cochrane to assess the quality of all included literature and ROB. The assessment includes: sequence generation; allocation concealment; blinding of participants, personnel, and outcome assessors; incomplete outcome data; selective outcome reporting; other sources of bias. The evaluation above would be independently evaluated by 2 researchers. If there are different opinions, we discuss them. If there are still differences exist, we would consult the third appraiser. Otherwise, we need to consult with the Cochrane Professional Group for solution.

#### 2.3.1 Assessment of heterogeneity

We will use a *χ*^2^ test to estimate heterogeneity of both the mean difference and OR. Further analysis can be performed using the *I*^2^ test. If possible, we will also construct a forest plot for analysis. A random-effect model will be used to interpret the results if heterogeneity is statistically significant, whereas a fixed-effect model will be used if heterogeneity is not statistically significant. We will regard heterogeneity as substantial when *I*^2^ is >50% or a low *P* value (<.10) is reported for the *χ*^2^ test for heterogeneity.

#### 2.3.2 Sensitivity analysis

We will conduct a sensitivity analysis to identify whether the conclusions are robust in the review according to the following criteria: sample size, heterogeneity qualities, and statistical model (random-effects or fixed-effects model).

#### 2.3.3 Publication bias

If a result of a meta-analysis contains >10 articles and above, we will use a funnel plot to test the risk of publication bias.

#### 2.3.4 Quality of evidence

The quality of evidence for the main outcomes will also be assessed with the Grading of Recommendations Assessment, Development and Evaluation approach. The evaluation included bias risk; heterogeneity; indirectness; imprecision; publication bias. And each level of evidence will be made “very low,” “low,” “erate,” or “high” judgment.

## 3 Discussion

Coronary heart disease angina pectoris is a common and frequently occurring disease in internal medicine, which has caused great harm to the lives and health of the people.^[21]^ In recent years, the published global death cause and disease burden analysis report pointed out that the number of global coronary heart disease deaths exceeded 7 million, ranking first among 235 single-cause deaths.^[22]^ The results of the study show that coronary heart disease is the second leading cause of death in the Chinese population. Standardized drug interventions and revascularization treatments can achieve certain effects. However, how to further improve the clinical efficacy, reduce the incidence of cardiovascular events, improve the clinical symptoms of patients, and improve the quality of life of patients, these problems are the key issues to be resolved in the treatment of coronary heart disease angina.^[23,24]^ Chinese medicine treatment of coronary heart disease angina, in addition to oral medication, there is acupuncture therapy. With the increasing side effects of drug use and drug resistance, acupuncture is becoming more and more popular.^[25]^ Acupuncture can significantly improve the vascular endothelial function and cardiac function in the animal model of myocardial ischemia in coronary heart disease, and effectively improve the blood lipid level and the expression of related proteins and inflammatory factors in coronary heart disease model animals, thus effectively delaying the progression of atherosclerosis.^[26]^ Evidence from basic animal studies supports the efficacy of acupuncture in the treatment of coronary heart disease with angina pectoris, and several clinical randomized controlled trials support this theory. Compared with conventional treatment, acupuncture can reduce the symptoms of angina pectoris in patients with coronary heart disease, improve electrocardiogram, reduce the amount of nitroglycerin, and improve hemodynamics.^[27]^

The specific mechanism of acupuncture for angina pectoris is unclear, so we will conduct a systematic review and meta-analysis to evaluate the efficacy and safety of acupuncture in the treatment of angina pectoris.^[28]^ The results of this study may provide a possible ranking for acupuncture treatment of angina. In addition, the scoring method will be used to assess the quality of the evidence for the primary outcome. We hope that these results will provide clinicians with the basis for acupuncture treatment of angina and provide the best choice for patient care. In addition, although this study will conduct a comprehensive search, it will not search for languages other than Chinese and English, which will lead to some bias.^[9]^

## Author contributions

**Data curation:** Sheng Deng, Xu-dong Yu

**Formal analysis:** Ji-sheng Wang, Xu-dong Yu

**Funding acquisition:** Sheng Deng, Hong-wei Yuan

**Project administration:** Hai-song Li, Ji-sheng Wang

**Supervision:** Ji-sheng Wang, XSY

**Validation:** Sheng Deng, Ji-sheng Wang, Xu-dong Yu

**Writing – original draft:** Hong-wei Yuan, Hai-song Li

**Writing – review & editing:** Hong-wei Yuan, Hai-song Li

## References

[1] Geng N Su G Wang S High red blood cell distribution width is closely associated with in-stent restenosis in patients with unstable angina pectoris[J]. BMC Cardiovascular Disorders 2019;19:10.1186/s12872-019-1159-3 PMC6651917 31340761

[2] Jing S Jian W Yuan W Correlation between referred pain region and sensitized acupoints in patients with stable angina pectoris and distribution of sensitized spots in rats with myocardial ischemia[J]. Acupunct Res 2018;43:277–84. 10.13702/j.1000-0607.180123 29888560

[3] Al-Janabi F Mammen R Karamasis G In-flight angina pectoris; an unusual presentation[J]. BMC Cardiovasc Disord 2018;18:61. 29699500 10.1186/s12872-018-0797-1 PMC5921980

[4] Ong P Sechtem U [optimal diagnostics and therapy for microvascular angina pectoris][J]. Dtsch Med Wochenschr 2017;142:1586–93. 29046002 10.1055/s-0043-104469

[5] Dose N Michelsen MM Mygind ND Ventricular repolarization alterations in women with angina pectoris and suspected coronary microvascular dysfunction[J]. J Electrocardiol 2017;51:10.1016/j.jelectrocard.2017.08.017 28939174

[6] Zengguang F Yabin Z Foot bath and yangxin decoction: clinical observation of angina pectoris of coronary heart disease[J]. Liaoning Journal of Traditional Chinese Medicine 2017.

[7] Liu KT Wang X Zhao D Effect of PCI on inflammatory factors, cTnI, MMP-9 and NT-pro BNP in patients with unstable angina pectoris[J]. Journal of Hainan Medical University 2016;22:

[8] Junxian W Cardiology DO Application of home-made trimethazine in unstable angina pectoris[J]. Clinical Medicine 2016.

[9] Wyckoff RT Zu Neuen Ufern, by Louis H. Lorenz[J]. Deutsche Zeitschrift Für Akupunktur 2012;55:10–4.

[10] Lun-Hui Y Bao-Hong M Jie C Clinical study about acupuncture combined with cupping therapy for treating stable exertional angina pectoris in the middle-age or old patients[J]. Chinese Journal of Clinical Healthcare 2009;12:465–7.

[11] Ruskin AP Sphenopalatine (NASAL) ganglion: its role in pain, spasm and the rage reaction and possible relationship to acupuncture[J]. Acupunct Electrother Res 1979;4:91–103.

[12] Potier JP Moineau D Orberk E [At every dialysis, this patient suffered angina pectoris]. Nephrol Ther 2017;13:103–4. 28159483 10.1016/j.nephro.2016.07.452

[13] Braumann S Bartram MP Pfister R [Angina pectoris in a young woman with lupus erythematosus][J]. Dtsc Med Wochenschr 2017;142:1449–52. 10.1055/s-0043-112074 28938508

[14] Yoshimura H Oba T Nagata T Unstable angina pectoris in a 22-year-old female patient[J]. Kurume Med J 2017;63:33–7. 28090005 10.2739/kurumemedj.MS6300008

[15] Fan H Zhao L Li J Strategy on the recruitment of free community medical-consultation in acupuncture clinical trials[J]. Zhongguo zhen jiu = Chinese acupuncture & moxibustion 2016;36:413. 27352507

[16] Elgendy IY Winchester DE Pepine CJ Experimental and early investigational drugs for angina pectoris[J]. Expert Opinion on Investigational Drugs 2016;25:10.1080/13543784.2016.1254617 PMC5228503 27791405

[17] Zhang Z Chen M Zhang L Meta-analysis of acupuncture therapy for the treatment of stable angina pectoris[J]. Int J Clin Expe Med 2015;8:5112–20. PMC4483941 26131084

[18] Wang N Lu SF Chen H A protocol of histone modification-based mechanistic study of acupuncture in patients with stable angina pectoris[J]. BMC Complement Altern Med 2015;15:139. 25925670 10.1186/s12906-015-0653-0 PMC4465012

[19] Wang M Chen H Lu S [Impacts on neutrophil to lymphocyte ratio in patients of chronic stable angina pectoris treated with acupuncture at Neiguan (PC 6)][J]. Zhongguo Zhen Jiu 2015;35:417–21. 26271132

[20] Miaomiao J Ying L Ruirui S Taking accupoints along the meridians to treat angina pectoris[J]. World Chinese Med 2015.

[21] Osler W Angina pectoris and arteriosclerosis[J]. JAMA 2015;314: 1981. 10.1001/jama.2014.12084 26547477

[22] Li ZJ Zeng F Lan L [Using functional brain imaging technique to study central mechanism of acupuncture therapy for chronic stable angina pectoris in view of heart-brain correlation].[J]. Zhen Ci Yan Jiu 2014;39:337–40. 25219133

[23] Wang M Acupuncture styles in current practice[M]. Translational Acupuncture Research 2019.

[24] Yuan W Wang Q Perioperative acupuncture medicine: a novel concept instead of acupuncture anesthesia[J]. Chinese Med J 2019;132:707–15. 10.1097/CM9.0000000000000123 PMC6416101 30855351

[25] Condello G Chen CY Fostering the acupuncture practice for health outcomes research: the perspective from Taiwan[J]. J Chin Med Assoc 2019;82:1. 31259834 10.1097/JCMA.0000000000000135 PMC13048092

[26] Spinka F Aichinger J Wallner E Functional status and life satisfaction of patients with stable angina pectoris in Austria[J]. BMJ Open 2019;9:e029661. 10.1136/bmjopen-2019-029661 PMC6731841 31488483

[27] Tzanis G Palmisano A Gallone G The impact of the coronary sinus reducer upon left ventricular function in patients with refractory angina pectoris[J]. Catheter Cardiovasc Interv 2019. 10.1002/ccd.28408 31373415

[28] Geng N Su G Wang S High red blood cell distribution width is closely associated with in-stent restenosis in patients with unstable angina pectoris[J]. BMC Cardiovasc Disord 2019;19:1000. 10.1186/s12872-019-1159-3 PMC6651917 31340761

